# Correction: Factors affecting patient satisfaction in refugee health centers in Turkey

**DOI:** 10.1371/journal.pone.0296371

**Published:** 2023-12-20

**Authors:** Monica Zikusooka, Radysh Hanna, Altin Malaj, Meliksah Ertem, Omur Cinar Elci

There is a mistake in the second author’s name. The correct name is: Hanna Radysh.

There is an error in the affiliation for Omur Cinar Elci. The correct affiliation is: Western Atlantic University School of Medicine, Freeport, Grand Bahama.
